# Adapted Lethality: What We Can Learn from Guinea Pig-Adapted Ebola Virus Infection Model

**DOI:** 10.1155/2016/8059607

**Published:** 2016-02-18

**Authors:** S. V. Cheresiz, E. A. Semenova, A. A. Chepurnov

**Affiliations:** ^1^Department of Medicine, Novosibirsk State University, Pirogova Street 2, 630090 Novosibirsk-90, Russia; ^2^Institute of Internal and Preventive Medicine, Bogatkova Street 175/1, 630089 Novosibirsk-89, Russia; ^3^Institute of Clinical Immunology, Yadrincevskaya Street 14, 630047 Novosibirsk-47, Russia

## Abstract

Establishment of small animal models of Ebola virus (EBOV) infection is important both for the study of genetic determinants involved in the complex pathology of EBOV disease and for the preliminary screening of antivirals, production of therapeutic heterologic immunoglobulins, and experimental vaccine development. Since the wild-type EBOV is avirulent in rodents, the adaptation series of passages in these animals are required for the virulence/lethality to emerge in these models. Here, we provide an overview of our several adaptation series in guinea pigs, which resulted in the establishment of guinea pig-adapted EBOV (GPA-EBOV) variants different in their characteristics, while uniformly lethal for the infected animals, and compare the virologic, genetic, pathomorphologic, and immunologic findings with those obtained in the adaptation experiments of the other research groups.

## 1. Introduction

Several animal models for Ebola virus infection have been established in rodents and nonhuman primates (NHPs). The NHPs, including rhesus and cynomolgus macaques, are best suited for pathogenesis, treatment, and vaccine studies, since only they can be lethally infected by nonadapted EBOV strains with the resulting pathology closely resembling the human EBOV disease [1]. However, due to ethical, practical, and expense reasons, small animal models of EBOV infection were developed including guinea pig, mouse, and, recently, Syrian hamster models [2]. Those are established by a serial passage required for virus adaptation, since the wild-type EBOV is avirulent or causes a nonlethal disease in rodents. Although even the lethal adapted EBOV infection in rodents is different in many aspects from the disease in primates, the important similarities in the courses of both infections make small animal models useful, especially, in the study of genetic determinants of EBOV disease and in antiviral screening [1].

In primates, the pathogenesis of EBOV infection is associated with the viral replication in several major cell targets accompanied with immune dysregulation and coagulopathies. Viral reproduction in primary targets, the mononuclear phagocytes of spleen and lymph nodes, is followed by a massive replication in the liver, mostly, in macrophages and hepatocytes, and the virus spread to the other organs and tissues (adrenals, kidneys, reproductive organs, and lungs). A bystander lymphocyte apoptosis by an unknown mechanism is proposed to be the cause of severe lymphopenia occurring in EBOV infection. Inhibition of IFN-mediated response mediated by viral proteins VP24 and VP35 blocks the innate antiviral defense. Vascular damage either occurring directly, due to lytic virus reproduction in the endothelial cells, or induced indirectly by the effects of proinflammatory cytokines on the vascular wall is an important factor of pathogenesis. The mechanisms of coagulation dysfunctions, such as disseminated intravascular coagulation (DIC) and hemorrhages, as well as thrombopenia occurring in primate EBOV infection, are still to be investigated in more detail [3].

In guinea pigs, the lethal EBOV variants are established through the sequential passages (4–8 times) of an originally wild-type virus, in which, first, incomplete and, further, complete lethality in the groups of inoculated animals are acquired [4–7]. The guinea pig-adapted EBOV is causing a lethal infection with minor manifestations in the first 4-5 days and a subsequent rapid development of a highly febrile condition resulting in the animal death on days 8–11. First detected in lymph node macrophages as early as 24 h p/i, the virus spreads to the spleen and liver on day 2 and to the other organs and tissues further on. The virus spread can be accompanied with a progressive rise of tissue virus titers (from 1.7 to 4.8–6.4 log_10_ PFU/g in different tissues including spleen, liver, adrenals, lungs, kidneys, and pancreas) on days 1–9 of the infection, and the peak viremia in blood is reached on day 7 with ~10^5^ PFU/mL [7]. However, in two of our adaptation experiments, an only modest [8] or even zero increase in virus titer [9] between the nonlethal and lethal adapted EBOV was occurring. A prolongation of the prothrombin time (PT) and the partial thromboplastin time (aPTT) is observed in the infected animals [1].

While resembling the course of EBOV infection in primates in many aspects, the EBOV disease in rodents has some important differences. Fever and maculopapular rash, which are the typical signs of infection in primates, are both not present in mice infected with mouse-adapted Ebola virus (MA-EBOV) [10]. In guinea pigs infected with guinea pig-adapted Ebola virus (GPA-EBOV), only fever, but not the rash, is present [5, 7]. Unlike in mice and similarly to Syrian hamster, lethal EBOV infection in guinea pigs induces serious coagulation abnormalities including the drop of platelets and an increase in coagulation time; however, fibrin depositions and disseminated intravascular coagulation (DIC) are not readily observed in these animals [2, 7, 11]. Occurrence of hemorrhages in EBOV disease in guinea pigs is still disputable: some researchers report that death of animals is not accompanied by the visible signs of hemorrhage [6]; however, we regularly observed typical hemorrhagic foci in the liver of, at least, part of the animals infected with GPA-EBOV in our experiments [12]. Despite the severe lymphopenia in the course of GPA-EBOV infection, lymphocyte bystander apoptosis, which is an important feature of infection in primates and mice, is not generally observed in guinea pigs [7].

Here, we present a comprehensive overview of the virologic, pathomorphologic, and hematologic data obtained in the EBOV adaptation experiments in guinea pigs (including four passage series performed in our laboratory) and discuss the value of those findings obtained in the rodent models for the general understanding of pathology of EBOV infection.

## 2. Establishment of Guinea Pig-Adapted Lethal Strains of Ebola Virus and the Study of Their Virulence and Virologic Characteristics

Serial passage of different viruses in animals or cell cultures can result in the selection of mutants with different reproduction characteristics and either higher or lower virulence, as compared to the original wild-type virus [13, 14]. Adult guinea pigs inoculated with the wild-type EBOV isolated from primates typically develop a nonlethal mild febrile or even asymptomatic disease. However, it has been previously shown that young guinea pigs may occasionally develop a lethal EBOV infection, and the serial passage of EBOV causes a substantial increase in lethality in these animals [4, 15]. In this review paper, we summarize our own data on the establishment of four independent adaptation series in guinea pigs using the original stocks of EBOV subtype Zaire, strain Mayinga, differing in their passage history [5, 8, 9, 11], and compare them with those from other labs where the EBOV adaptation series were successfully performed [6, 7, 10].

The original EBOV, strain Mayinga, stock of the first adaptation series (8mc) was passaged 24 times in Vero E6 cell line prior to the guinea pig adaptation experiments [5], while the stock of the second series (K-5) was passaged twice in NHPs (green monkeys) followed by 15 passages in human lung L68 cells [9]. In the third series (GLA), the wild-type EBOV, strain Mayinga, was passaged twice in NHPs and further multiplied in Vero cell culture [11]. The fourth series (GPA-P7) was started with an individual clone of strain Mayinga EBOV, which was obtained by triple-cloning/passaging in Vero cells and was confirmed to cause no lethal disease prior to its adaptation to guinea pigs. This virus clone further used for the inoculation of animals was designated as the precursor of guinea pig-adapted EBOV (pre-GPA clone) [8].

At first passage of 8mc adaptation series [5], a group of animals (we used the groups of 6 animals in all our adaptation experiments) was inoculated with virus derived from a liver homogenate of a rhesus macaque lethally infected by Mayinga strain of EBOV and passaged 24 times in Vero E6 cells. In some animals, a nonlethal febrile condition developed with the body temperature rising up to 40°C. The liver homogenates of the animals, which demonstrated high-grade fevers and higher levels of liver virus titer, were used for the next passage inoculation. With each passage, the febrile temperature was increasing (from 39.8–40.0°C at earlier passages to 41.5–42.0°C at later passages), as was the liver virus titer, which showed the overall growth from 2.9 ± 0.4 log_10_ PFU/mL to 5.5 ± 0.7 log_10_ PFU/mL (as determined by plaque forming assay in Vero cells). The first lethal outcomes were detected at passage 3, and the lethality and infectivity of the adapting virus isolate were gradually increasing until passage 8. In guinea pigs inoculated with the virus isolates of late passage 8, the fever occurred at days 4–7 and represented a double-peaked pattern with the second peak at days 11–13. Fever episodes were associated with the maximum levels of tissue virus titers. The adapted virus strain was designated EBOV-8mc, and several clones were derived from it and proved to have similar features as the noncloned EBOV-8mc virus itself [5].

As mentioned above, the original EBOV stock of K-5 series was passaged twice in NHPs and further 15 times in L68 cell culture prior to its adaptation to guinea pig, and its infectivity and virulence were evolving in the passage series. The biological titer of the virus harvest in PFU decreased from the first to third passage and then began to increase, reaching its plateau by passage 12 and further remaining stable. However, the virus infectivity in guinea pigs was unchanged throughout the L68 passages. Following the cell culture passages, the virus cloning was performed, and the biological titer (in PFU) of the intermediate virus stock (after passage 15, stock designation Ch-15) and the three obtained clones were shown to be equal. When adaptation to guinea pigs was started, clone 2 turned out to cause no febrile reaction, and virus isolation from blood samples of inoculated animals failed. However, clone 1 caused a rise in the rectal temperature in guinea pigs up to 40.5–41.8°C after the first passage, affecting up to 100% of animals. Beginning from the fourth passage, lethal outcomes were recorded. After the fifth passage, the virus became highly lethal for guinea pigs. The virus presence in the blood was recorded at a titer of 3.5 log_10_ PFU/mL at passage 1 and did not change throughout the passage series. The resulting virus strain was designated K-5 [9].

In the third passage series, GLA [11], the animals inoculated at passage 1 developed a low-grade (below 40°C) fever, which showed a double-peak pattern with the peaks at days 7 and 20. A low amount of virus (1.62 log_10_ PFU/mL) could only be detected in the serum after the virus concentration by centrifugation, while no virus could be isolated from the whole blood. The tissue virus titers were detectable at 1.6 log_10_ PFU/mL (in liver) and 3.22 log_10_ PFU/mL (in peritoneal exudates). The first lethal outcomes were detected at passage 2 (on day 10), concomitant with the increase of serum and tissue virus titers to 4.2 log_10_ PFU/mL. Interestingly, the second peak of fever was not observed in the surviving animals. At passage 3, the adapting EBOV became fully lethal to the guinea pigs with all animals dying between days 5 and 7, and the serum and tissue virus titers stabilized at the levels of 3.0–4.0 log_10_ PFU/mL. The GPA-EBOV strain obtained in this series was used for a comprehensive analysis of pathomorphologic, hematologic, immunologic, and coagulation patterns of EBOV disease in guinea pigs.

In the last series, GPA-P7 [8], the precursor pre-GPA clone was obtained by a triple passage of original Mayinga EBOV in Vero cells and subsequent cloning. In the course of adaptation series, at each passage, one animal that displayed the most pronounced temperature response by day 7 p/i was used as a donor of the virus for the next passage. At all passages, the fever onset in the infected animals was recorded on day 4 p/i. The duration of fever episodes increased from passage to passage (from 6 days at passages 1-2 to 10–12 days in the surviving animals at passages 5-6). By day 7 p/i, the animals of all passages displayed a low level of viremia, which was not increasing throughout the passage series. The ability of virus to reproduce in the liver of infected animals was not changing significantly, as well. In the first two passages, EBOV induced a mild disease in guinea pigs with an increase in temperature to 40.0–40.4°C on days 4–9 p/i and eventual recovery. The first lethal outcome was recorded at passage 3, at day 15 p/i. No lethal outcomes were recorded at passage 4, while two lethal outcomes (40% lethality) were recorded at passage 4. Further on, the lethality increased, while the time of survival decreased. At passage 6, 80% lethality was observed, and, finally, at passage 7, full lethality of infection was acquired. The survival time in this fully lethal EBOV infection averaged 11.6 days. In lethal infections, the animals rapidly lost weight, displayed anorexia, and developed impaired motor coordination, especially in the terminal stage. Two-three days before death, guinea pigs had diarrhea and the signs of intestinal hemorrhage were observed. This highly lethal EBOV variant obtained by the 7-passage adaptation of pre-GPA clone was designated GPA-P7 [8].

Our several attempts were made to adapt two original Mayinga strain EBOV stocks (of adaptation series 8mc and K-5) to adult ICR mice, using the same approach as the adaptation series to guinea pigs. All attempts to adapt the K-5 virus stock failed; however, using high doses of the first EBOV stock, we obtained a paradoxical variant of EBO. This passage series in adult ICR mice caused no lethal cases but resulted in the increasing virus titer in the liver reaching 5.55 × 10^11^ PFU/mL at later passages. Of note, the EBOV titers in the liver even at the first passage reached 3.5 × 10^9^ PFU/mL on day 11. This mouse-adapted virus variant was named D-5 [9].

**Figure 1:**
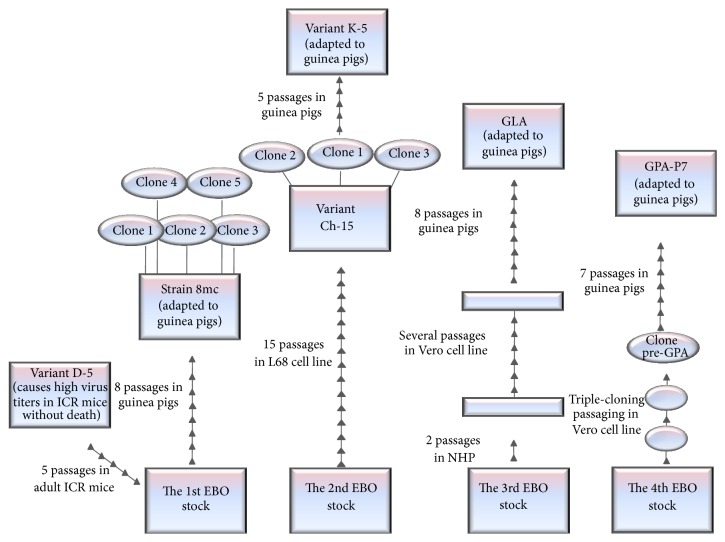
Adaptation series of wild-type Mayinga strain EBOV to guinea pigs and mice resulting in the establishment of 8mc, K-5, GLA, and GPA-P7 lethal guinea pig-adapted virus variants and a paradoxical high-titer, avirulent mouse-adapted virus variant D-5.

All adaptation series and the designations of all original and derivative viral stocks are provided in Figure 1.

The guinea pig-adapted EBOV strains, 8mc, K-5, GLA, and GPA-P7, as well as two other adapted variants obtained by the other research groups [6, 7], thus, represent the virus variants, which appear to be very similar in their virulence and lethality (despite the number of passages needed to acquire the full-scale pathogenicity), while strikingly different in the serum and tissue virus titers in the course of adaptation. Two of our adaptation series, 8mc and GLA, and the two series reported by others [6, 7] represent a 2-3-order-of-magnitude growth of tissue virus titers (in PFU) accompanying the development of strain virulence for guinea pigs, while in the other two, K-5 and GPA-P7, no or slight virus titer increase has been observed despite the acquisition of full lethality to the animals [5, 8, 9]. The adaptation series [5–7, 11], in which an increase in the virus titers in the liver or spleen has been observed (Table 1), prompted the hypothesis that the development of high virulence/lethality of the adapted virus variants could be due to the increasing virus replication in the infected tissues. However, the establishment of K-5 and GPA-P7 virus stocks, which do not show any significant increase in virus titers, while demonstrating high lethality to the animals, does not support this hypothesis. Similarly, a proposed link between the increasing rate of virus replication and the development of lethality in the adaptation of Mayinga EBOV strain to mice [10] is contradicted by our data on the mouse-adapted D-5 variant demonstrating the extraordinary high tissue virus titers, while lacking any pathogenicity in ICR mice [9]. It seems, therefore, that an increase in virus reproduction may not be the central determinant of EBOV virulence evolution in the course of virus adaptation to different animal models.

## 3. Study of the Genetic Determinants of Virulence/Lethality in Guinea Pig-Adapted EBOV Variants

The information on the genetic markers of EBOV virulence is important for the understanding of EBOV infection pathogenesis, as well as for the development of vaccines and antivirals. Since some new mutations (as compared to the wild-type virus stocks) can occur* de novo* and some rare mutations can be amplified and fixed in the adapted virus stocks in the process of adaptation, comparison of genetic changes in adapted lethal and preadaptation nonlethal EBOV genomes and matching of those to the virulence of different EBOV variants can yield invaluable information on the genetic determinants of virulence/lethality.

In our first adaptation series, 8mc, a detailed analysis of nucleotide sequences of the EBOV genomes of the original and guinea pig-adapted virus stocks revealed several changes in genome regions encoding for the five viral proteins [9, 16]. The 8mc clones showed, in total, eight common sequence changes compared to the original first EBO stock. Two silent base changes were found in the ORF encoding NP protein, which are, obviously, not affecting the NP function, while another base change in NP gene leads to a conservative amino acid substitution Phe→Leu(648), also unlikely to affect the virulence. No base changes were found in vp35 and vp40 genes. A single uridine insertion at the gene-editing site of viral glycoprotein (GP) gene and an amino acid substitution Asp→Gly(397) were found in three of five 8mc clones; however, all five clones were similar in their pathogenicity for guinea pigs, despite the significant change of GP expression pattern caused by the former mutation. A single base change in the 3′ noncoding region of vp30 gene also seems irrelevant to the acquisition of virus pathogenicity in the course of adaptation.

Contrarily to the above mutations, at least some of the three nucleotide changes in the coding region of vp24 gene, which led to amino acid substitutions Met→Ile(71), Leu→Pro(147), and Thr→Ile(187), may be linked to the adaptation process, since the gel mobility of 8mc-EBOV VP24 is lower than its wild-type counterpart, suggesting a major structural rearrangement of this protein and, probably, the crucial role for VP24 in the adaptation process.

A conservative amino acid substitution Thr→Ala(820) in L gene, similarly to Phe→Leu(648) substitution in NP gene, does not seem to be responsible for virus virulence. However, since both are found in all five EBOV-8mc clones, they may have a supportive role in the increase in virulence [16].

A comparative nucleotide sequence analysis of the virus variants of the second adaptation series (K-5) reveals two single nucleotide substitutions in vp24 ORF in the adapted virus strain K-5 (as compared to the original second EBOV stock), which lead to the amino acid substitutions Asp→Gly(165) and His→Tyr(186) [17]. Since the former is present in the initial virus variant Ch-15 used for adaptation in guinea pigs, it has, obviously, been introduced during passaging in NHPs or L68 cells and seems irrelevant to the development of pathogenicity of an adapted virus. The other vp24 mutation is found at nearly the same location in EBOV variant K-5, as in another guinea pig-adapted variant, 8mc, obtained in the first passage series, thus, supporting the hypothesis of a possible involvement of this protein in the host adaptation and increase in pathogenicity [16].

To further evaluate the contribution of genetic differences present in guinea pig-adapted EBOV variants in the virus virulence, two different approaches were employed. The first one was another adaptation series started with a cloned EBOV stock (pre-GPA), in which the genetic events occurring were determined at every passage and compared to the pathogenic reactions in guinea pigs reflecting the emerging EBOV virulence [8]. The other study was using the reverse genetic approach and was focused on the effects of individual mutations of EBOV-8mc variant and their combinations on EBOV virulence [18].

In the former study, the GPA-P7 adapted strain genome was found to differ from the genome of the pre-GPA clone by 17 nucleotide changes. No genetic differences were found in the terminal untranslated regions of the GPA-P7 genome (3′-leader and 5′-trailer). The nucleotide sequence of the GPA-P7 NP gene contained 12 nucleotide substitutions, 8 of those being synonymous and 4 leading to amino acid changes in the encoded NP protein (Leu→Pro(544), Asn→Ser(566), Ser→Pro(598), and Asn→Asp(663)). Three substitutions leading to amino acid changes were detected in the nucleotide sequence of the GPA-P7 VP24 gene (Met→Ile(71), Leu→Pro(147), and Arg→Leu(154)). The L gene (encoding the RNA-dependent RNA polymerase) of GPA-P7 contained two nucleotide substitutions, one synonymous and one nonsynonymous, the latter resulting in amino acid change (Val→Ile(236)) [8].

Correlation of the mutations in GPA-P7 with the time of their occurrence (passage number) and the emerging virus pathogenicity in guinea pigs allows one to evaluate their roles in the virulence. Of 17 nucleotide substitutions that distinguish between GPA-P7 and the pre-GPA clone, 12 occurred as early as the first passage (GPA-P1 variant). These 12 substitutions comprise nine synonymous (eight in NP gene and one in L gene) and three nonsynonymous substitutions resulting in 3 amino acid changes in NP gene (Leu→Pro(544), Ser→Pro(598), and Asn→Asp(663)). Those mutations were considered not related to the emerging EBOV virulence, since they occurred before the acquisition of lethality. Two other mutations emerged at passage 3, leading to the amino acid changes Asn→Ser(566) in NP protein and Leu→Pro(147) in VP24 protein. Of note, the first lethal outcome in this series was recorded at passage 3, although no lethal outcomes among the infected animals were recorded at passage 4. Those two loci remained heterogenous until the substitutions were fixed in the genome of the GPA-P5 variant (at passage 5) representing increased virulence for guinea pigs and causing the disease with 40% lethality (2 lethal outcomes of five observed animals). At passage 6, a mutation in the VP24 gene sequence, G10557A, occurred resulting in amino acid change Met→Ile(71). This passage was accompanied by a further increase in EBOV virulence for guinea pigs, appearing as an 80% lethal disease (four lethal outcomes between days 10 and 12 out of 5 animals). At passage 7, a nucleotide substitution G10805U, resulting in an amino acid change Arg→Leu(154), was fixed in the VP24 gene sequence, and a heterogeneous site (G12286A, Val→Ile(236)) appeared in the L gene sequence. Emergence of these mutations correlated with a further increase in EBOV pathogenicity for guinea pigs, which manifested as 100% lethality (between days 8 and 12). Thus, only the three amino acid changes in VP24 protein (positions 147, 71, and 154), one change in NP protein (position 566), and one change in L-polymerase (position 236) may have an essential contribution to the gradually emerging virulence of EBOV in the course of GPA-P7 series [8]. Of interest, two identical mutations in VP24 (positions 71 and 147) were discovered in the abovementioned 8mc adapted virus strain [16].

In the latter study [18], recombinant viruses carrying various combinations of wild-type and guinea pig-adapted genes were generated by reverse genetic approach and their contribution to the EBOV virulence in guinea pigs was evaluated. The membrane-associated protein VP24 was shown to play a critical role in EBOV pathogenesis, and amino acid changes in VP24 were shown essential and sufficient to confer EBOV virulence in guinea pigs. Indeed, recombinant virus stocks carrying only those 8mc mutations harbored in VP24 gene are causing a uniformed lethality in guinea pigs. Inoculation of animals with the viruses carrying NP and/or L gene mutations did not show any sign of the disease except for the death of two guinea pigs in the group infected with recombinant virus carrying a mutation in NP gene (rEBOV-NP/8mc, F648L). Sequence analysis of virus from the blood of those dead animals revealed the additional amino acid substitution L26F in the VP24. Inoculation of guinea pigs with virus from such animals resulted in their uniform death. To evaluate the role of this mutation in the virulence of guinea pig-adapted EBOV, a recombinant virus was generated that differed from the wild-type virus by a single mutation in VP24, rEBOV-VP24/L26F. Strikingly, all animals infected with rEBOV-VP24/L26F developed signs of EBOV disease approximately 4 days p/i. Importantly, no additional mutations were found in the viruses recovered from guinea pigs inoculated with this recombinant virus, suggesting that adaptive mutations in VP24 are necessary and sufficient for an increase in EBOV virulence in guinea pigs. The “8mc” mutation in NP (Phe→Leu(648)) is not strictly required for the increase in pathogenicity but seems to lead to the accelerated selection of EBOV variant, in which a single mutation in VP24 was able to confer lethality [18].

Of interest, a similar major contribution of VP24 mutations in the EBOV adaptation to mice has been observed, with the requirement for the supportive mutations in NP [19]. The role of VP24 and NP proteins in the virulence of mouse-adapted EBOV strain, however, correlated with their ability to evade IFN-I-triggered antiviral responses. The guinea pig-adapted and wild-type EBOV VP24, on the contrary, were similar in their ability to block IFN-I-stimulated response [18], which underlines the importance of different animal-adapted EBOV models in the dissection of a complex phenotype of EBOV disease.

A summary of all genetic determinants revealed in the guinea pig adaptation series of EBOV is provided in Table 2.

## 4. Pathomorphological Findings in EBOV-Infected Guinea Pigs

Ebola virus infection in primates is associated with diffuse parenchymal necrosis in the liver without marked inflammatory changes [20]. Unlike that, Ryabchikova and colleagues report the occurrence of granulomatous lesions in the liver to be the hallmark of the wild-type EBOV infection in guinea pigs [6]. Those granulomas are proposed to play an important role in virus clearance. Granuloma-like foci consisting of the monocytes, macrophages, and neutrophils appear at day 5 p/i and increase in size and number thereafter. No involvement of hepatocytes was observed in this study until days 7-8, when some of the perifocal hepatocytes demonstrate lipid droplet accumulation and destruction of organoids. The other organs were only slightly involved, except for the spleen and lymph nodes, in which some signs of immunosuppression (decreased number of mitoses, lack of plasma cell formation, and destruction of stroma cells and macrophages) were found. Virus reproduction occurred in the mononuclear phagocytes of the liver only at later stages of infection (on day 7) and was observed as the presence of viral nucleocapsids and specific membranous structures in the infected cells. Most of the infected macrophages were located in the granuloma-like foci and contained virions, which appeared eroded with their nucleoids indistinct. No reproduction of wild-type virus was detected in the other organs [21].

**Figure 2:**
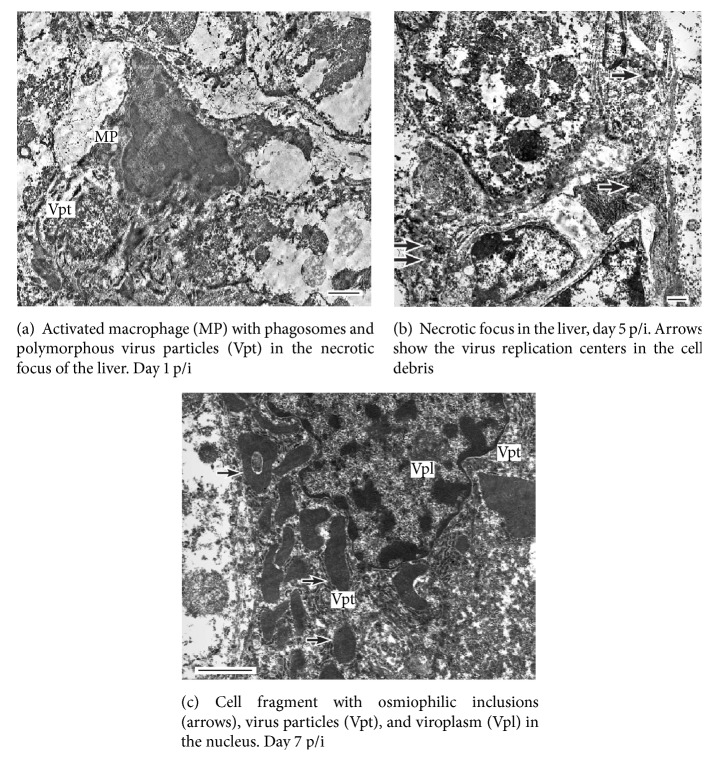
Wild-type EBOV nonlethal infection in guinea pigs. Electron microscopy of the liver.

In our study, we were able to observe the dynamics of pathomorphologic changes in wild-type EBOV infection in guinea pigs [12], which was somewhat different from the pattern described above. Electron microscopy revealed the occurrence of EBO virions in the dilated Disse spaces as early as 24 h p/i. Simultaneously, a developing inflammatory reaction in the periphery of liver acini was observed with ballooning degeneration of hepatocytes showing disorganization of organelles (swollen mitochondria) and necrotic foci containing phagocyting macrophages and virus particles (Figure 2(a)). However, no virus replication centers were observed in the macrophages. By day 5, the necrobiotic changes are progressing and involve the major part of liver parenchyma with the often occurrence of necrotic hepatocytes, numerous hemorrhages in the swollen subendothelial space, and centers of virus replication in the necrotic foci, although with only few virus particles visible (Figure 2(b)). By day 7 of infection, however, the progression of necrobiotic changes ceases, and the tissue structure stabilizes. At this time, macrophages with numerous osmiophilic inclusions, nuclei containing viroplasm, and numerous virions in their neighborhood are seen (Figure 2(c)). The major difference from the course of wild-type EBOV infection in guinea pigs observed by Ryabchikova et al. [6] was the presence of damaged hepatocytes at days 5–7 p/i.

The gradual emergence of EBOV virulence to guinea pigs in a series of passages is reflected in the changes of pathomorphological pattern of the infection. At early passages (1–3), only quantitative differences in the numbers of activated and infected liver macrophages were observed. The pathomorphological pattern of infection changes at passage 4, with greater numbers of liver macrophages involved, as well as the virus reproduction first detected in hepatocytes. However, only the formation of nucleocapsids, not the proper virus budding, occurs in hepatocytes at this stage, making it unclear whether the infection in these cells is productive. In general, the liver lesions are more severe than at earlier passages, and the signs of spleen and kidney involvement become evident. All observed morphological changes gradually become more prominent at passages 5-6, with liver focal lesions becoming widespread and number of infected cells and virions increasing. With the acquisition of full lethality of the adapted virus to guinea pigs (passage 8), the hepatic tissue becomes filled with infected hepatocytes and macrophages with intracellular nucleocapsids, as well as numerous virions. Some cells contain structurally abnormal viroplasm and nucleocapsids. Areas of liver necrosis containing lysed hepatocytes, macrophages, and virions become all-pervading. Other organs and tissues show greater severity of lesions, as well [21].

**Figure 3:**
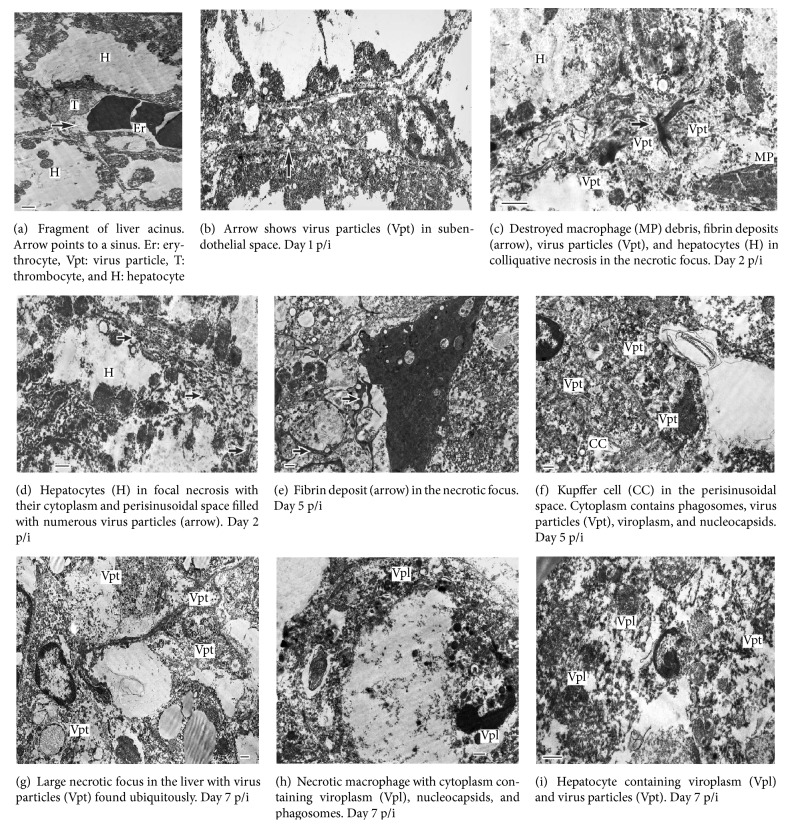
GPA-EBOV lethal infection in guinea pigs. Electron microscopy of the liver.

In GPA-EBOV-infected guinea pigs, we, similarly, observed the necrotic hepatocytes (Figure 3(a)) and virus particles in subendothelial space (Figure 3(b)) as early as 24 h p/i [12]. On day 2, the tissue destruction is more prominent, with multiple hemorrhages and necrotic foci containing numerous virus particles, fibrin deposits, and macrophages (Figure 3(c)). The centrilobular hepatocytes, typically, show the signs of ballooning dystrophy, while the perilobular hepatocytes become necrotic and contain virus particles (Figure 3(d)). This pattern indicates a progression of liver damage on day 2. By day 5, the necrotic process involves the central parts of acini where numerous necrotic foci with fibrin impregnation (Figure 3(e)) and Kupffer cells with viroplasm and virus particles (Figure 3(f)) are detected. At day 7, the pronounced and overall destruction of liver tissue of animals infected with GPA-EBOV is visible with large areas of necrosis involving both stromal and parenchymal cells (Figure 3(g)). Those necrotic lesions contain actively phagocyting macrophages with small and large phagosomes, viroplasm, and virus particles (Figure 3(h)), as well as hepatocytes containing numerous viruses (Figure 3(i)). Also, by this time, the liver lesions involve massive hemorrhages resulting in the occurrence of new areas of inflammation and the generalization of liver injury. We believe that the lethality in GPA-EBOV-infected guinea pigs is, thus, due to the triggering damage by the virus and the subsequent involvement of different areas of the liver resulting from the inflammatory reaction [12].

Day-by-day dynamics of pathomorphologic changes occurring in the GPA-infected guinea pigs illustrated by Connolly and colleagues [7] is in a good agreement with structural changes described above. In animals infected with the adapted, fully lethal EBOV variant, the viral RNA and antigens are detected in the lymph node macrophages as early as 24 h p/i. The number of infected macrophages, in which the typical EBOV intracytoplasmic inclusions representing viral nucleocapsids are seen by electron microscopy, increases 4–20-fold by 48 h p/i, confirming an ongoing productive infection. From day 3, a developing multifocal lymphoid necrosis and the depletion of lymph nodes of different localization are observed, which progressed by days 7–9. Ultrastructurally, the sinuses contain necrotic cellular debris, infected macrophages, and free virions, while no evidence of EBOV-infected lymphocytes in the lymph nodes is found. In the spleen, the occurrence of single-cell necrosis in the red pulp is seen at day 2 p/i, progressing into a more extensive necrosis of both splenic white and red pulp and the depletion of hematopoietic elements in the red pulp by day 4. All signs of productive infection (viral antigens, RNA, and intracytoplasmic inclusions) are detected in the red pulp macrophages and the antigen-presenting cells (dendritic and reticular cells) of some white pulp follicles. The spread of infection is associated with numerous circulating monocytes and tissue macrophages containing EBOV inclusions, with some of infected cells already showing budding virions and clusters of virions along their plasma membrane. In the liver, small foci of hepatocellular necrosis occur at days 2-3 p/i with EBOV-staining hepatocytes and Kupffer cells seen. On day 4, the ongoing infection in the liver spreads to the hepatocytes surrounding the necrotic foci and Kupffer cells, since they also begin to contain viral cytoplasmic inclusions. Most of these foci stain EBOV RNA- and antigen-positive, and the sinuses are filled with antigen-positive debris. Free virions in the Disse spaces and virus capsid inclusions are found in Kupffer cells at day 5. Later, at day 7, some periportal fibroblast-like cells become involved in the infection and stained EBOV-positive. The occurrence of the virion-containing fibrin depositions in subendothelial spaces and virus particles in subepithelial connective tissue of the bile ducts (both as free virions and cytoplasmic viral inclusions in degenerate fibroblasts), probably, marks the onset of a systemic spread of infection from the liver at day 9. In fact, at later stages of infection, involvement of different organs and tissues, as well as cellular targets, is observed: gastrointestinal fibroblasts and macrophages, adrenal cortical cells and fibroblasts, bladder epithelium, interstitial and alveolar macrophages, the occasional fibroblasts and endothelial cell of pulmonary venules in the lungs, and so forth [7, 21].

An independent morphological study of the wild-type and guinea pig-adapted EBOV infection reveals an inability of the nonadapted virus to reproduce in its primary target, the macrophages [18]. Peritoneal guinea pig macrophages infected* in vitro* by the recombinant virus obtained by reverse genetics and carrying the adaptive mutations in VP24 identical to those occurring in the 8mc adaptation series show characteristic inclusion bodies containing viral nucleocapsids. Contrarily, no typical viral nucleocapsids but only massive protein inclusions were found in the cells infected by the wild-type virus, which correlated with the lack of infectious virus release from those cells. This data suggested an efficient block of wild-type EBOV infection already in the primary target cells, probably, at the stage of VP24-mediated nucleocapsid assembly/budding [18].

Thus, the morphologic patterns of wild-type and adapted EBOV infection in guinea pigs are suggestive of the inability of the virus to establish productive infection in macrophages and, particularly, in hepatocytes. Failure to massively produce infectious wild-type EBO virions may result in the host defense successfully blocking further virus spread by locking the infection in granuloma-like inflammation foci with a subsequent virus clearance. The adaptive mutations in the EBOV genome can restore the ability of a virus to reproduce in its major targets, macrophages and hepatocytes, thus, facilitating systemic virus spread in the infected animals that results in high virulence. On the other hand, this mechanism of adaptation would present itself as the increase of blood and liver virus titers in the adapted virus-infected animals, as compared to those inoculated with the wild-type virus, which is not the case for two adaptation series, K-5 and GPA-P7. There should be, therefore, an alternative mechanism of virulence increase during adaptation, which is relevant to those series. A morphological study of K-5 and GPA-P7 virus infections, which has not been done as yet, and its comparison with the morphological patterns of infection described earlier would, thus, be of interest.

## 5. Hematologic, Immunologic, and Coagulation Shifts in the Guinea Pig Infections by Wild-Type and Adapted Virus

Wild-type EBOV infection causes serious hematologic aberrations in its natural hosts including leukocytosis due to marked neutrophilia and, frequently, lymphopenia in terminally ill primates. Thrombocytopenia develops in acutely ill human patients and experimentally infected primates experiencing hemorrhagic syndrome. Unlike that, both neutrophilia and lymphopenia can be observed in EBOV-infected guinea pigs as early as day 2 p/i and are progressing over the course of the disease. According to some reports, the scale of those hematological shifts in individual infected animals can be as great as a >3.5-fold increase in the number of neutrophils and >3.0-fold drop in the number of lymphocytes. This finding appears intriguing, since lymphocytes are not infected by EBOV* in vivo* despite the progression of lymphoid necrosis, and they are resistant to infections by different EBOV strains* in vitro* [7]. The bystander lymphocyte apoptosis, an important feature of EBOV infection in primates and mice, has not been determined in guinea pigs infected with adapted EBOV, as well [2]. The mechanisms of lymphoid necrosis and depletion occurring both in primates and in guinea pigs remain unclear but are, probably, caused by the events resulting from a massive macrophage infection and changing the microenvironment of lymphoid tissue. Severe thrombocytopenia (>14-fold, as compared to the preinfection levels) develops later, on day 7 of infection (two days before death at day 9), resembling the loss of platelets in terminally ill primates [7].

It appears, though, that the leukocyte counts should be taken with care, since a wide-range variation of different leukocyte types between the animals and their experimental groups is natural to guinea pigs (cf. neutrophil/lymphocyte percentage in the reported group, 21/77, to the data from one of our adaptation series, 57/38 [11]). Therefore, the analysis of dynamics of hematologic indices at different passages of adaptation and its correlation with the clinical course of EBOV disease appears to be more valid.

In one of our adaptation series, GLA, we specifically aimed to study the evolution of different blood indices in the course of adaptation [11]. In this experiment, the leukocyte counts remained stable throughout passages 1–3, demonstrating a significant 3-fold decrease at next passage 4, remaining at this lower level throughout the further adaptation. This passage was also notable for the first occurrence of eosinophils in blood samples and an abrupt (~8-fold), although transient, increase in monocyte and plasmacyte counts, which returned to baseline values at the next passage. Also, the amount of neutrophils dropped by 20% at this passage (transiently, to recover at passage 5), while the percentage of young, atypical, and large, probably activated, lymphocytes showed a transient increase at passage 4 (also to return to the baseline values at passage 5). Interestingly, these changes followed the acquirement of full lethality by the adapting EBOV at passage 3 [11].

The percentage of young thrombocytes demonstrated a 3-fold increase at passage 2 and an overall 12-fold increase by passage 6. The total thrombocyte count remained stable until it fell abruptly (2-fold) at passage 6. The occurrence of young thrombocytes at early passages, before the fully lethal EBOV has been selected, indicates the important shifts in the thrombocytic hemostasis, which resulted in a progressive loss of platelets observed in the next passages.

An interesting finding was the change of erythrocyte morphology in the EBOV adaptation. The percentage of normal red blood cells was progressively decreasing at every passage concomitant with the occurrence of echinocytes and erythrocytes with inclusions, which suggests the exit of immature erythrocytes from the bone marrow, apparently, to compensate for the deficiency of red blood cells in the organism. The occurrence of echinocytes in the peripheral blood has been noticed in the acute viral hepatitis and some other viral and inflammatory diseases [22]; however, the involvement of erythrocytes into the EBOV infection has never been reported earlier.

The shifts of the peripheral blood counts were accompanied by the changes in bone marrow composition, firstly, by the increase in the number of blast cells as early as passage 2, reaching its peak of 65 pro mille by passage 5 but further dropping. This may indicate compensatory activation of bone marrow function with subsequent exhaustion. Megakaryocyte counts started growing at passage 2 and reached their peak at passage 5, clearly reflecting the upward dynamics of young thrombocytes.

Finally, the phagocytic function of neutrophils was progressively decreasing from passage 2, although the number of neutrophils was variable at different passages. Contrarily, the phagocytic function of peritoneal macrophages was remaining stable for 6 passages and then dropping abruptly [11, 23].

Although many of the hematologic indices were fluctuating in the passage series or changing at later passages (which allowed ruling out their causative role in the emerging lethality of adapting EBOV), some were evidently concomitant with the increasing pathogenicity or even appearing prior to the acquiring of full lethality by the adapted virus. Those include the increase of the percentage of young thrombocytes along with the growing number of blast cells and megakaryocytes and the decrease of phagocytic activity of neutrophils, all commencing as early as passage 2, alongside the significant growth of blood and tissue virus titer and the occurrence of the first lethal cases in the inoculated animals. It is, thus, tempting to hypothesize that the evolving wild-type EBOV acquires new characteristics triggering those early responses in hematologic parameters [11]. Those key parameters were also changing consistently in our other adaptation series, GPA-7, although it was rather different from the above GLA experiment, since the virus titer was not growing in the passage series and the full lethality has been acquired later, at passage 5 versus passage 3 for GLA series [8]. However, the decreasing phagocytic activity of neutrophils was also an early response appearing at passage 2, and the trend of accumulation of atypical lymphocytes in the course of adaptation was evident, probably, due to the progressing apoptotic and necrotic processes.

A rather intriguing immunologic finding was the lack of correlation between the blood and peritoneal TNF levels and the pathogenicity of EBOV infection in experimental animals. The guinea pigs infected with wild-type Mayinga or lethal adapted 8mc EBOV demonstrated similar, rather low, levels of TNF activity in blood and no TNF activity in peritoneal exudates (Table 3). Contrarily, when rabbits, which are completely resistant to EBOV infection, were inoculated with a wild-type EBOV, a rather high serum TNF activity was detected early (day 3 p/i) and was progressively increasing to extraordinary high values later on (days 5 and 9 p/i). In rabbits, TNF activity was also detected in peritoneal exudates (Table 3) [23]. The previously published data report the elevated proinflammatory cytokine levels in the lethal wild-type EBOV infections in humans and nonhuman primates [24, 25] and in baboons infected with guinea pig-adapted EBOV. High levels of TNF-*α* production by peripheral blood macrophages infected* in vitro* by another filovirus, Marburg virus, were proposed to be the major pathogenetic factor of Marburg virus infection due to its ability to induce hemorrhagic shock [26]. Thus, our data on TNF activity in the rodent models of EBOV infection (guinea pigs and rabbits) are contradictory to the proposed causative role of TNF-induced hemorrhages in primate infections.

Since coagulopathies, such as disseminated vascular coagulation (DIC) or massive hemorrhages, are the hallmark of EBOV disease in primates [27] and considering the tight and clinically relevant cross talk between the coagulation and complement systems [28, 29], it seems important to discuss the role of complement involvement in the experimental EBOV infection in guinea pigs.

**Figure 4:**
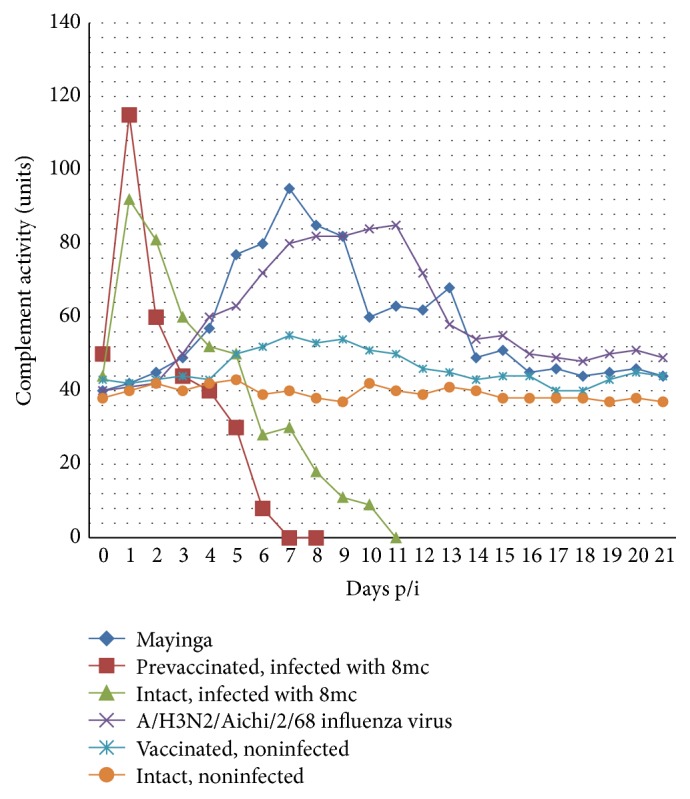
Complement activity in the intact and prevaccinated guinea pigs infected with lethal GPA-EBOV-8mc strain or nonlethal wild-type EBOV Mayinga strain. Animals infected with nonlethal influenza virus A/Aichi/2/68 (H3N2) strain, intact animals, and guinea pigs vaccinated with inactivated nonlethal Mayinga strain EBOV while not infected were used as different controls of complement activity. Slow lagging dynamics of complement activity in guinea pigs infected with nonlethal Mayinga strain EBOV is contrasting to the rapid onset/drop peak of complement activity in the intact and prevaccinated animals infected with a lethal 8mc strain GPA-EBOV.

The dynamics of complement activity in wild-type EBOV-infected guinea pigs and in the intact or EBOV-preimmunized animals infected with a lethal adapted EBOV strain demonstrates striking differences, which can be important for the understanding of EBOV-induced coagulopathies. In wild-type EBOV-infected animals, the complement activity shows a double-peak profile with a 2-day lag, the first, higher peak at days 4–7 and second, lower peak at days 9-10. Contrarily, in animals infected with the adapted lethal 8mc strain, the complement activity demonstrates a rapid peak at 24 h p/i followed by an abrupt decline on day 2, return to the baseline by days 4-5, and further drop to the background levels by days 9–11, followed by the animal death. Finally, the guinea pigs preimmunized with formalin-inactivated EBOV and infected by a lethal 8mc strain showed even a more steep rise of complement activity on day 1 and a more abrupt fall on day 2 p/i with a further decline to near-zero values by day 6, also followed by the animal death by day 8 (Figure 4).

The rate of complement activity growth in the lethally infected guinea pigs suggests the involvement of an alternative pathway of complement activation. In preimmunized animals, the classic activation can additionally occur, due to the presence of anti-Ebola immunoglobulins, which may be the possible cause of higher complement activities than in nonimmunized animals. Alternatively, slower kinetics of complement activation in guinea pigs infected with nonlethal strain indicates classic activation with the two peaks corresponding to IgM (before day 7) and IgG production (days 7–14). Rapid complement activation by alternative pathway appears to be a unique ability of the 8mc lethal EBOV strain to immediately hyperactivate complement production with subsequent fast and complete exhaustion of its synthesis [30]. Importantly, the retrospective study of the sera from days 1 and 5 and later of a single fatal case of a laboratory staff EBOV infection accident reveals the similar pattern of a sharp increase and an immediate drop of complement activity, suggesting the relevance of this data for humans [12].

Interestingly, the pattern of serum concentrations of circulating immune complexes (CICs) is closely resembling the kinetics of complement activity both in intact and in preimmunized lethally infected animals, with a typical rapid rise in the first day and an immediate decline to near-zero values by days 8-9 or 5-6 in intact or preimmunized animals, respectively, followed by the animal death. Slow CIC and complement kinetics are also very similar in the nonlethal EBOV infections of guinea pigs. The significance of circulating immune complexes (CICs) in the pathogenesis of Ebola disease still remains to be studied [30].

Since no coagulation abnormalities are occurring in the mouse-adapted EBOV model, which is a general feature of mouse models for acute viral infections [19], the guinea pig-adapted EBOV remains a unique nonprimate model for the study of EBOV-induced coagulopathies.

## 6. Conclusion

Since the first isolation of wild-type EBOV strains, the problems with the animal model for the study of biology and therapy/prevention of this infection became evident. Resistance of the most convenient rodent model animals (mice, guinea pigs, hamsters, and rabbits) to EBOV infection prompted the researchers to use adaptation of wild-type EBOV strains to the small animal models. Practically, the EBOV adaptation to guinea pigs was the easier and the first to be achieved. The lethality of the guinea pig-adapted EBOV variant to the nonhuman primates remained unchanged [31]. In the adaptation passage series of EBOV stocks differing in their passage histories described here and previously, the emergence of the fully lethal GPA-EBOV variants was readily achieved in 7 passages or less. EBOV adaptation to mice required much more efforts (M. Bray, personal communication). In our own attempt of EBOV adaptation to mice, a paradoxical nonlethal strain was obtained, which demonstrated high-level replication and persistence of virus in the animal's liver, while lacking any clinical symptoms of the disease. The historically first GPA-EBOV strain designated 8mc was originally established for the vaccine development and heterologic immunoglobulin production [32]. However, it was soon realized that this variant not only represents a convenient small animal model, but also offers the opportunity to compare the genetic differences between the wild-type and adapted EBOV strains and establish the correlation between the genetic determinants and the emerging and evolving virulence and lethality in a new host [16]. Also, the comparison of pathomorphologic, immunologic, hematologic, and coagulation features of EBOV infection by the wild-type and adapted virus allows one to establish the most important physiologic parameters, which influence the course of EBOV disease [9, 11, 12, 23].

We and others were able to demonstrate the mutations in EBOV VP24 protein to be the most important determinants of the virus virulence and lethality [8, 9, 16, 18].

The need to reproduce the consistency of processes occurring in the course of adaptation and establish the details of adaptation process required the repeated adaptation series starting from the virus stocks with different passage histories including the cloned EBOV stocks.

The differences in the dynamics of hemolytic complement activity found in the infections of guinea pigs by the wild-type and adapted EBOV strains allowed us to be able to predict the outcome of EBOV infection in the first hours p/i [30].

Also, our studies indicate strong suppression of neutrophilic phagocytosis in the infections of guinea pigs with GPA-EBOV strain, while only its mild activation on day 5 of wild-type EBOV infection was observed. This suggested that the reproduction of EBOV in macrophages, on the one side, with concomitant suppression of neutrophils, on the other, results in the disturbance of neutrophil/macrophage interaction as a driving force of a lethal EBOV disease.

Another interesting discovery was the presence of young thrombocytes in GPA-EBOV-infected animals. The phenomenon, which was lacking in the wild-type EBOV-infected animals, together with the increase of megakaryocyte counts in the bone marrow, can be the evidence of the activation of megakaryocytic hematopoiesis and the exit of nonmature platelets into the circulation.

Previously, the elevated levels of proinflammatory cytokines have been reported to occur in the course of EBOV disease and contribute to the infection lethality in humans and NHPs, due to their role in the development of EBOV coagulopathies. However, in guinea pigs we, paradoxically, found higher (while comparable) TNF-*α* levels in the animals infected with a nonlethal wild-type than in those infected with lethal adapted EBOV, thus, demonstrating lack of TNF involvement in GPA-EBOV pathogenesis.

## Figures and Tables

**Table 1:** Comparison of lethality, virus reproduction, and the time to adaptation (# of passages) in different adaptation series.

Adaptation series	8mc [5]	K-5 [9]	GLA [11]	GPA-P7 [8]	[6]	[7]	D-5 [9]	[10]

Original virus stock	Mayinga, ZEBOV	Mayinga, ZEBOV	Mayinga, ZEBOV	Mayinga, ZEBOV	Mayinga, ZEBOV	Mayinga, ZEBOV	Mayinga, ZEBOV	Mayinga, ZEBOV

Preadaptation history	Vero E6 cell line passages	NHPs + L68 cell line passages	NHPs + Vero cell line passages	Individual clone, obtained from infected Vero cells	Vero E6 cell line passages	—	—	—

Adaptation to	Guinea pig	Guinea pig	Guinea pig	Guinea pig	Guinea pig	Guinea pig	Mouse	Mouse

Adapted virus lethality	100%	100%	100%	100%	80%	100%	0%	100%

Number of passages to full lethality	8	5	3	7	7	4	5	9

Increase in virus titer (nonadapted nonlethal versus adapted lethal, log_10_⁡ PFU/mL)	2.9/5.5 Liver	3.5/3.5 Plasma	0.0/4.2 Plasma 1.62/4.2 Liver 3.22/4.2 Peritoneal exudate	6.3/7.0	2.9/5.0	2.1/5.2 Plasma 1.7/(4.8–6.4) In different tissues	9.5/11.7	4.2/7.8

Note: all virus titers in our adaptation series 8mc, K-5, GLA, GPA-P7, and D-5 were determined on day 6 p/i.

**Table 2:** Genetic changes occurring in different adaptation series and reverse genetics experiments and their relevance to the development of GPA-EBOV virulence/lethality.

Viral ORF	Nucleotide position	Amino acid substitution	Relevance to adaptation	Comment
*EBOV-8mc adaptation series*				
NP	1852	None	No	Synonymous
	2410	None	No	Synonymous
	2411	Phe → Leu(648)	Unlikely	Conservative, may play a supportive role in adaptation
GP	6924 (insertion)	Frameshift	No	Clones with or without those GP mutations do not differ from each other in their pathogenicity in guinea pigs
	7228	Asp → Gly(397)	No	
VP30	9595	None	No	Located in 3′-untranslated region
VP24	10557	Met → Ile(71)	Probably	At least, one of those three VP24 mutations induces major structural rearrangements in EBOV-8mc VP24, which are presented as gel mobility shift in electrophoresis
	10784	Leu → Pro(147)	Probably	
	10904	Thr → Ile(187)	Probably	
L	14038	Thr → Ala(820)	Unlikely	Conservative, may play a supportive role in adaptation

*EBOV-5K adaptation series*				
VP24	10838	Asp → Gly(165)	No	Is acquired in the course of passage in NHPs or L68 cells, prior to adaptation
	10907	His → Tyr(186)	Probably	Acquired during adaptation, lies close to EBOV-8mc VP24 Thr → Ile(187)

*EBOV-GPA-P7 series*				
NP	1781	Asn → His(438)	No	Is found in nonadapted pre-GPA clone
	2043	Arg → Lys(525)	No	Is found in nonadapted pre-GPA clone
	2092	None	No	Synonymous
	2100	Leu → Arg(544)	No	Occurred at passage 1 after pre-GPA, before the acquisition of lethality
	2164	None	No	Synonymous
	2166	Asn → Ser(566)	Probably	Occurred at passage 3, when the first lethal outcome was recorded, heterogenous site until fixed at passage 5, concomitant with 40% lethality
	2188	None	No	Synonymous
	2191	None	No	Synonymous
	2209	None	No	Synonymous
	2222	None	No	Synonymous
	2233	None	No	Synonymous
	2260	None	No	Synonymous
	2261	Ser → Pro(598)	No	Occurred at passage 1 after pre-GPA, before the acquisition of lethality
	2409	Ser → Phe(647)	No	Is found in nonadapted pre-GPA clone
VP40	2456	Asp → Asn(663)	No	Occurred at passage 1 after pre-GPA, before the acquisition of lethality
	5576	None	No	Synonymous
VP24	5597	None	No	Synonymous
	10557	Met → Ile(71)	Probably	Occurred at passage 6, concomitant with lethality increase to 80%
	10784	Leu → Pro(147)	Probably	Occurred at passage 3, when the first lethal outcome was recorded, heterogenous site until fixed at passage 5, concomitant with 40% lethality
	10805	Arg → Leu(154)	Probably	Occurred at passage 7, concomitant with full lethality
L	12286	Val → Ile(236)	Probably	Occurred at passage 7, concomitant with full lethality
	16821	None	No	Synonymous

*Reverse genetics of EBOV-8mc mutations*				
VP24	10422	Leu → Phe(26)	Definitely	Derived from the blood of an animal, which died after inoculation with a nonlethal recombinant rEBOV-NP/8mc, F648L virus. When introduced into an otherwise wild-type Zaire EBOV genome by reverse genetics, this mutation alone confers full lethality to guinea pigs

**Table 3:** Serum and peritoneal TNF activity in EBOV-infected guinea pigs and rabbits.

Virus	Guinea pigs		Rabbits
	Mayinga (wild-type)	8mc, guinea pig-adapted	Mayinga (wild-type)
TNF activity, serum (U/mL)	3.4 ± 0.1 (day 7)	2.5 ± 0.16 (day 7)	104 ± 36 (day 3)
			471 ± 137 (day 5)
			1398 ± 909 (day 9)

Virus titer, serum (PFU)	10^3^	10^4^–10^5^	Nondetectable

TNF activity, peritoneal (U/mL)	Nondetectable	Nondetectable	15.3 ± 2.1 (days 5–9)

Virus titer, peritoneal (PFU)	10^2^–10^3^	10^2^–10^3^	Nondetectable
